# Neurocysticercosis mimicking eclampsia in a pregnant woman with a history of preeclampsia: a case report

**DOI:** 10.3389/fmed.2026.1900625

**Published:** 2026-08-04

**Authors:** Miguel Ochoa-Andrade, André Urdiales, Omar Morocho, Mateo Rojas, Aileen Salinas, María José Toro, Daniel Vergara-Pacheco, Jimmy Rivera-Álvarez, Geovanny Quishpe, Freddy Saldarriaga-Mera

**Affiliations:** 1 Medicine Program, School of Health Sciences, Universidad Técnica Particular de Loja, Quito, Ecuador; 2 Medicine Program, School of Health Sciences, Pontificia Universidad Católica del Ecuador, Quito, Ecuador; 3 Gynecology and Obstetrics, Medical Service, General Coordination of Maternal and Child Health, Hospital General del Sur de Quito, Quito, Ecuador; 4 Medical Program, School of Health Sciences, Universidad San Gregorio de Portoviejo, Portoviejo, Ecuador

**Keywords:** eclampsia, neurocysticercosis, parasitic diseases, preeclampsia, pregnancy

## Abstract

**Introduction:**

Eclampsia is the default suspected diagnosis in cases of seizures during pregnancy; atypical or treatment-resistant cases warrant consideration of differential diagnoses for neurological causes. In endemic Andean regions, neurocysticercosis, a brain infection caused by Taenia solium larvae, is one of the leading causes of acquired epilepsy. This case demonstrates how this parasitic infection can mimic a severe obstetric emergency.

**Case presentation:**

This case describes a 35-year-old mestiza woman at 26.1 weeks of gestation with a history of preeclampsia in two previous pregnancies. She presented to the emergency department with headache, epigastric pain, blurred vision, and normal vital signs, followed by several tonic-clonic seizures. Given this presentation, the medical team immediately suspected eclampsia and initiated the magnesium sulfate protocol, as its mechanism of action promotes cerebral vasodilation and reduces vasospasm, thereby favoring neuroprotection. Laboratory tests showed a urine protein/creatinine ratio of 0.58, with no abnormalities in hepatic or renal function parameters, and no recorded hypertensive values. Despite anticonvulsant administration, the seizures were not controlled, prompting neuroimaging studies. These studies revealed multiple cysts with scolices in the brain, confirming neurocysticercosis in the vesicular stage. Through a multidisciplinary approach, seizures were controlled with levetiracetam, allowing the pregnancy to reach term at 37.5 weeks. The patient had an uncomplicated normal vaginal delivery. In the postpartum period, she successfully started antiparasitic treatment with albendazole and dexamethasone.

**Conclusions:**

This case offers a valuable lesson on the diagnostic challenge posed by the overlapping neurological symptoms of eclampsia and neurocysticercosis. If a pregnant woman presents with atypical seizures or fails to respond to magnesium sulfate, neuroimaging is essential: it is a vital tool for ruling out structural abnormalities and ensuring the wellbeing of both mother and baby.

## 1 Introduction

Preeclampsia affects between 3% and 8% of pregnancies and, together with hypertensive disorders of pregnancy, accounts for up to 16% of maternal deaths worldwide, in addition to numerous fetal and perinatal complications (1, 2). It is a complex multisystem disease defined as hypertension >140/90 mmHg appearing after 20 weeks of gestation, associated with proteinuria and/or maternal organ dysfunction (2, 3). Although its etiology has not yet been fully elucidated, the clinical importance of preeclampsia lies in its complications, including placental abruption, HELLP syndrome, and eclampsia, entities associated with high maternal and perinatal morbidity and mortality (4–7).

Eclampsia is defined as the convulsive manifestation of hypertensive disorders of pregnancy, characterized by *de novo* focal or multifocal tonic-clonic seizures in the absence of another neurological etiology that explains the clinical presentation. Clinically, it may manifest with headache, visual disturbances, epigastric pain, and seizures, representing a major cause of maternal and fetal morbidity and mortality due to the risk of hypoxia, aspiration pneumonia, cerebral edema, fetal distress, and prematurity. Although classically associated with hypertension and proteinuria, up to 20%−38% of patients may not present these findings before the seizure, requiring consideration of neurological, infectious, structural, and vascular differential diagnoses, especially in atypical or recurrent presentations. Definitive resolution of hypertensive disorders of pregnancy occurs only after delivery (8).

Neurocysticercosis is a neurological differential diagnosis for seizures during pregnancy. It is a parasitic infection of the central nervous system caused by the larval stage of Taenia solium, considered the most common neurological helminthiasis worldwide and one of the leading causes of acquired epilepsy in developing countries. The cysticercus can invade the central nervous system, causing parenchymal lesions associated with seizures, as well as ventricular and subarachnoid lesions that can lead to hydrocephalus, intracranial hypertension, or vascular events (9–11). Transmission is associated with poor hygiene, low-technology pig farming, and consumption of water or food contaminated with the parasite's eggs.

In Ecuador, neurocysticercosis has been reported mainly in rural highland regions and agricultural areas, particularly in Andean provinces where conditions favorable to the parasite's transmission cycle persist (9). Factors such as poverty, overcrowding, limited access to potable water, and close contact with pigs are important predisposing factors. In this context, the coexistence or clinical confusion between eclampsia and neurocysticercosis is an infrequent but clinically relevant presentation because of the diagnostic and therapeutic implications that can affect the timely management of the pregnant patient (12).

## 2 Case presentation

This study presents the case of a 35-year-old mestiza woman, residing in Quito, Ecuador. She is currently in her fifth pregnancy, at 26.1 weeks of gestation at the time of initial admission. Obstetric history includes four previous cephalovaginal deliveries, with a history of preeclampsia in two prior pregnancies, one diagnosed at 39 weeks and the other at 20 weeks of gestation, both with favorable maternal-fetal outcomes.

The patient presented to the emergency department with a 2-h history of severe headache (9/10 on the VAS scale), epigastric pain, and blurred vision. Vital signs were within normal range. Hours later, she experienced two generalized tonic-clonic seizures: the first at home while sleeping, and the second during a bus ride, after which she was transported back to the emergency department by paramedics. She reported no relevant medical history or known drug allergies. She also reported no prior neurological history, such as epilepsy, and no significant family medical history.

On admission, the patient was conscious, alert, and oriented after the generalized tonic-clonic seizures. She presented with dysarthria, tongue biting, spontaneous urination, and signs suggestive of recent seizure activity. Proteinuria was also detected on urine dipstick testing.

The general physical examination revealed no signs of respiratory distress or significant hemodynamic instability. Cardiopulmonary evaluation was normal, with rhythmic heart sounds and adequate lung ventilation. Obstetric evaluation revealed a gravid abdomen with a uterine height appropriate for gestational age, a single live fetus in cephalic presentation, right dorsal position, a fetal heart rate of 138 beats per minute, and fetal movements, with no uterine activity at the time of examination. Pathological examination of the placenta showed no significant histological abnormalities, with findings consistent with a placenta of normal characteristics for gestational age.

Relevant clinical findings included severe headache, epigastric pain, blurred vision, and photophobia in the context of a 26.1-week pregnancy, which initially suggested a hypertensive disorder of pregnancy. The patient was hospitalized for continuous neuromonitoring due to the risk of seizure recurrence. The patient's immunological response was assessed from mid-pregnancy, correlating it with the gestational weeks at diagnosis.

**Figure 1:**
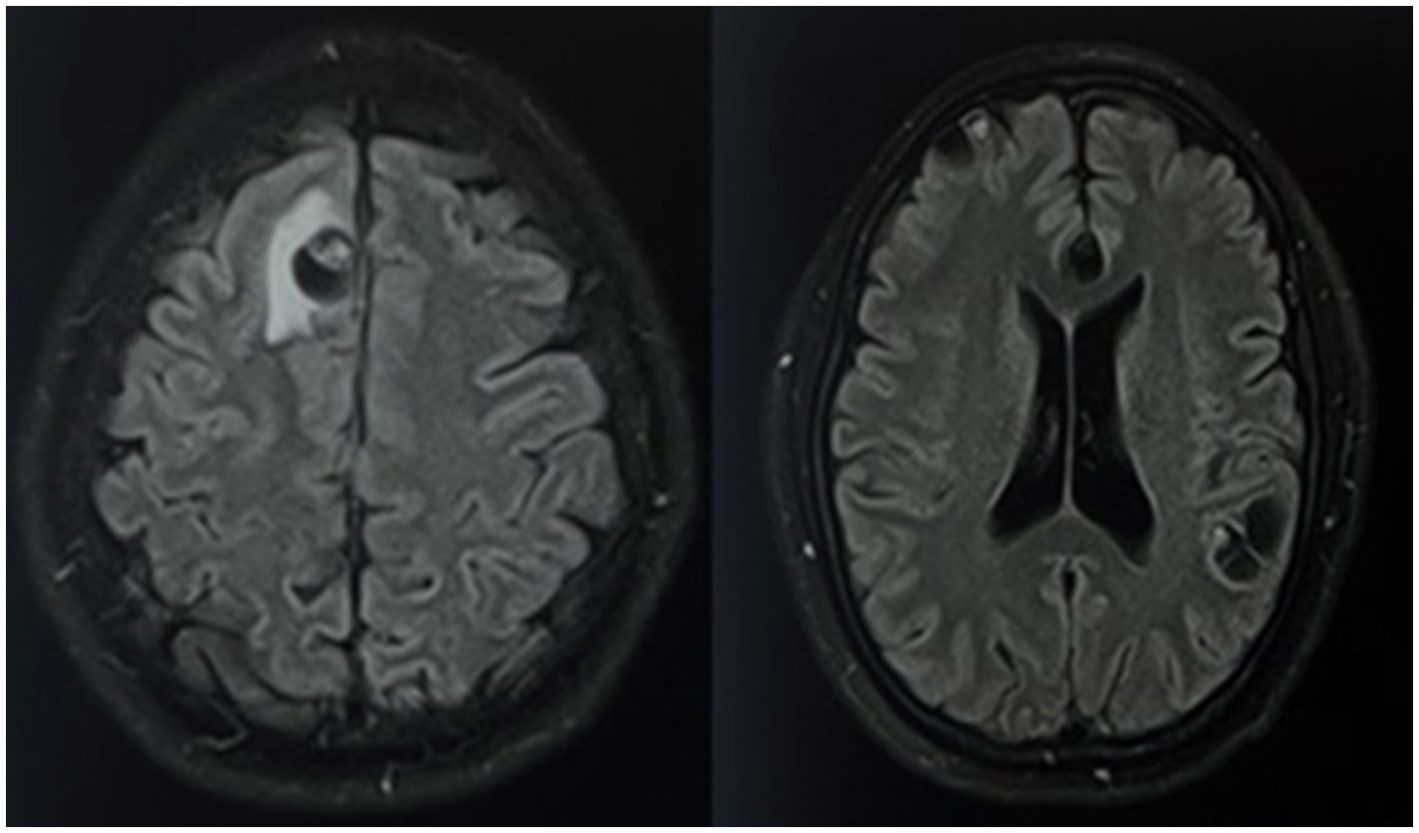
Non-contrast brain MRI – sagittal T1-weighted sequence.

**Figure 2:**
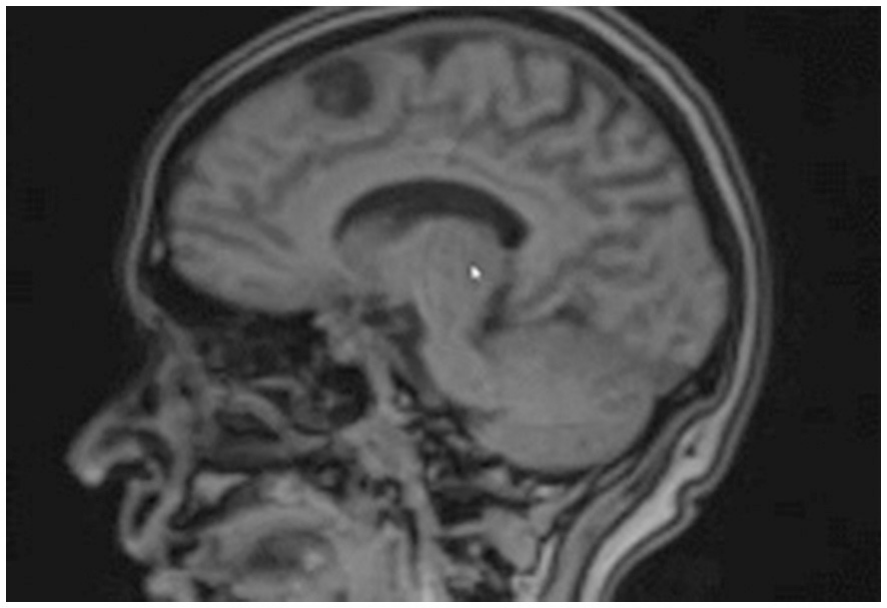
Non-contrast brain MRI – axial and coronal T2-weighted sequence.

**Figure 3:**
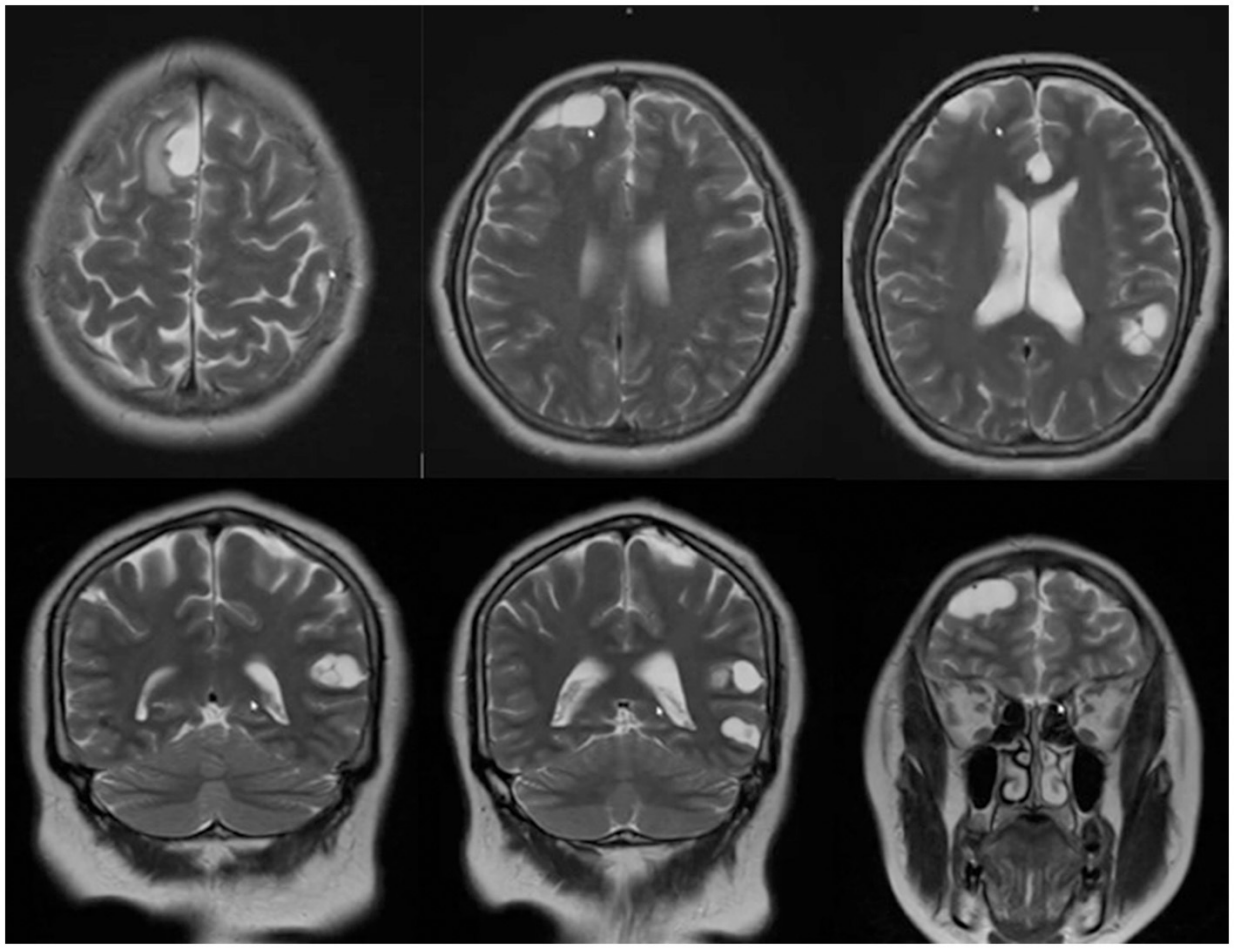
Non-contrast brain MRI – axial FLAIR sequence.

Due to the recurrence of seizures and the persistence of neurological symptoms, neuroimaging studies were performed. Non-contrast brain CT showed multiple intra-axial cystic and calcified lesions, consistent with neurocysticercosis at different evolutionary stages, with no mass effect or hydrocephalus. Subsequently, brain MRI confirmed the presence of multiple parenchymal cystic lesions with visible scolices, a finding highly suggestive of neurocysticercosis in the vesicular and colloidal vesicular stages. The lesions were distributed across different brain regions, predominantly in the right frontal, superior parietal, and bilateral occipital regions. The ventricular system was found in the midline, with preserved morphology and volume. On conventional MRI sequences, the lesions showed hypointense signal on T1 and hyperintense signal on T2 in the cerebral parenchyma, including the subcortical and cortical regions of the right frontal region. FLAIR suppression, no diffusion restriction, and hyperintensity on the ADC map were present, features consistent with cystic content. Some lesions showed a hyperintense halo on FLAIR and peripheral diffusion restriction, associated with perilesional vasogenic edema, findings consistent with lesions in the colloidal vesicular stage (Figures 1–3).

During obstetric follow-up, the postpartum period was uncomplicated, with a normal vaginal delivery and a newborn in good general condition; however, prolonged hospitalization was required. The newborn was delivered by cephalovaginal delivery at 37.3 weeks of gestation by last menstrual period (38 weeks by Capurro method). Adequate adaptation to extrauterine life was observed, with Apgar scores of 8 and 9 at the first and 5th min, respectively. Birth weight was 3,223 g, length was 48 cm, and head circumference was 36.8 cm. The neonatal physical examination was completely normal, with no evidence of congenital malformations or neurological abnormalities. During hospitalization, the newborn remained hemodynamically stable, without respiratory distress (oxygen saturation >94% on room air), with adequate suction, feeding tolerance, and successful initiation of breastfeeding within the first hour of life, allowing rooming-in with the mother. No immediate neonatal complications attributable to intrauterine exposure to levetiracetam or maternal disease were identified. Routine pediatric follow-up was indicated, with monitoring of growth and neurodevelopment during the first years of life, due to prenatal exposure to antiepileptic drugs and the maternal history of epilepsy secondary to neurocysticercosis.

During the mother's multidisciplinary in-hospital follow-up, a favorable clinical course was observed, with no new seizure episodes after optimization of anticonvulsant treatment and initiation of antiparasitic therapy, in addition to corticosteroid and dexamethasone therapy to control perilesional edema. The patient remained hemodynamically stable, with no neurological deterioration or evident focal deficits, and with progressive improvement of her headache.

At postpartum follow-up, the patient progressed favorably, with no recurrence of seizures and no new neurological deficits. Physical examination was normal, with adequate puerperal involution and no obstetric complications. Given the diagnosis of multilesional parenchymal neurocysticercosis in the vesicular stage, etiological treatment with albendazole combined with corticosteroids in a tapering regimen was started. In contrast, antiepileptic treatment with levetiracetam 1,000 mg orally every 12 h was maintained. The patient continued joint follow-up with the Neurology and Obstetrics departments, with good treatment adherence and adequate clinical progress. Recommendations were provided regarding warning signs, treatment adherence, and periodic outpatient follow-up visits to monitor the clinical response and promptly detect seizure recurrence or complications related to neurocysticercosis.

The clinical, diagnostic, and therapeutic course of the case is presented in Table 1.

**Table 1:** Timeline of historical and current information on the evolution of the presented case as evaluated by the medical professional.

Moment/time	Clinical event	Relevant finding	Diagnosis	Interventions performed	Evolution
Initial care	Intense headache and initial neurological symptoms	Headache VAS^ᴥ^ 9/10, epigastric pain, blurred vision	Headache associated with common cold	Paracetamol IV and PO^*^	Partial improvement
Early morning day 01	First seizure at home	Generalized tonic-clonic seizure during sleep	Suspected neurological event	Transferred by family member	Persistent symptoms
Transfer to hospital	Second seizure	Seizure on bus, postictal state, and tongue biting	Recurrent seizure	Treated by paramedics	Postictal patient
Admission	Initial maternal-fetal evaluation	Intense headache, epigastric pain, photophobia, and proteinuria ++	26.1 weeks of gestation, suspected preeclampsia	Clinical evaluation and monitoring	Stable
Re-evaluation	Confirmed seizures	Pregnancy-related seizures and proteinuria	Suspected diagnosis of eclampsia	Admission and monitoring	Stable
Key diagnosis	Seizures in pregnant woman	Seizures associated with pregnancy and proteinuria	Eclampsia	Magnesium sulfate for neuroprotection	Seizure control
Monitoring	Maternal-fetal evaluation	Maternal-fetal monitoring	26 weeks pregnancy + eclampsia	Continuous patient monitoring	Normal fetal heart rate
Laboratory studies	Renal evaluation and proteinuria	UPCR^∞^ 0.58 positive, 24-h proteinuria 223.88 mg	Elevated proteinuria	Renal/hepatic profile	No severe damage
Imaging: brain CT^β^ scan	Structural neurological findings	Multiple cystic and calcified lesions without mass effect.	Neurocysticercosis	Neurology consultation	Diagnosis clarified
Brain MRI^α^	Etiological confirmation	Vesicular lesion with scolex and perilesional edema	Multilesional parenchymal neurocysticercosis + pregnancy	Multidisciplinary management	Diagnosis confirmed, stable

^*^IV, Intravenous; PO, Oral; ^ᴥ^VAS, Visual Analog Scale; ^β^CT, Computed Tomography; ^**α**^ MRI, Magnetic Resonance Imaging; ^∞^(UPCR), Urine Protein-Creatinine Ratio.

## 3 Discussion

Neurocysticercosis is a zoonosis caused by T. solium, whose definitive hosts are humans and pigs. The disease originates from the accidental ingestion of eggs or cysticerci, which subsequently lead to invasion. Thus, intestinal taeniasis occurs when a person consumes raw or undercooked pork containing cysticerci, which develop in the human small intestine into adult tapeworms. In contrast, cysticercosis/neurocysticercosis occurs when a person directly ingests the parasite's eggs through fecal-oral contamination or autoinfection. The released oncospheres penetrate the intestinal wall, migrate hematogenously, and lodge mainly in muscle and brain tissue, where they form larval cysts. While the cyst remains viable, it generally elicits little inflammation; when it degenerates or dies, it triggers edema, inflammation, and subsequent calcification. The disease presents with manifestations due to the parasite's mass effect, an inflammatory response, and chronic sequelae such as fibrosis, granulomas, and calcifications (9–11).

Interestingly, during pregnancy, maternal immunological changes favor a more tolerant immune environment, which could promote the survival of viable cysticerci and, in theory, limit the inflammatory response associated with the lesions. However, certain pregnancy-specific local immunological and physiological changes could influence the onset or recurrence of seizures. Although there is no evidence of vertical transmission of Taenia solium to the fetus or of teratogenic effects, prolonged seizures represent a significant risk due to the possibility of maternal-fetal hypoxia, fetal distress, and preterm delivery (10–12).

The onset of seizures in a pregnant patient from the Quito region, Ecuador, with a personal history of preeclampsia in two of her four previous pregnancies and a prodromal picture of severe headache, epigastric pain, blurred vision, and proteinuria, constitutes a clinical presentation that almost identically mimics eclampsia (8). The overlap of symptoms required a critical diagnostic approach and thorough exclusion, ruling out the following entities:

Eclampsia was the initial suspected diagnosis due to proteinuria and the history of preeclampsia. However, it was definitively ruled out after confirming the absence of blood pressure values in the severe range (hemodynamic stability on admission) and, pathognomonically, by neuroimaging findings showing multiple cystic lesions with visible scolices, incompatible with the pathophysiology of eclamptic vasospasm (13).

Posterior Reversible Encephalopathy Syndrome is a clinically plausible entity given the headache, photophobia, and visual disturbances. On MRI, this syndrome is characterized by symmetric vasogenic edema, predominantly in the parieto-occipital white matter regions (13). Although the patient showed a hyperintense FLAIR halo in these regions, it was neither confluent nor diffuse, but rather multifocal, microcystic, and strictly perilesional in distribution (edema satellite to the colloidal vesicular stages), which ruled out this entity.

Cerebral venous thrombosis: The intrinsic hypercoagulable state of pregnancy increases the risk of this pathological entity, which can present with severe headache, visual impairment, and seizures (14). This condition was rigorously excluded because the MRI sequences (especially T1, T2, and FLAIR) demonstrated that the ventricular system was centered in the midline, with preserved morphology and volume, and with intact patency of the dural venous sinuses, ruling out the empty delta sign or non-lobar focal hemorrhagic infarcts (15).

Neuroinfections (bacterial or fungal meningoencephalitis, tuberculoma, or toxoplasmosis: The absence of febrile syndrome, neck stiffness, or signs of permanent focal neurological deficit made an acute meningeal infection less likely. Likewise, toxoplasmosis or tuberculoma lesions typically show a thick, irregular ring-enhancement pattern with central necrosis; in this case, visualization of the scolex (the “dot-in-the-cyst” sign) and complete suppression of the cystic content on FLAIR confirmed the purely parasitic etiology (15, 16).

### 3.1 Atypical presentation of the case

Given this suspicion, brain CT and MRI studies revealed findings consistent with multilesional parenchymal cerebral neurocysticercosis associated with perilesional edema, identified as the underlying structural cause of the seizures. Despite the parasitic burden, the absence of mass effect, hydrocephalus, hemorrhage, or permanent focal deficits suggested a favorable neurological prognosis following the initiation of multidisciplinary anticonvulsant and antiparasitic management.

Considering the epidemiological context of Ecuador, where neurocysticercosis is endemic (particularly in rural Andean regions), this case exhibits markedly atypical features that challenge the usual clinical and radiological patterns of the disease during pregnancy (8, 9).

Simultaneous multilesional parenchymal form: Classically, manifestations of neurocysticercosis during pregnancy are associated with solitary lesions or isolated granulomatous reactivations due to protective immunotolerance (4, 10). The presence of multiple lesions at different evolutionary stages (vesicular and colloidal vesicular), distributed concomitantly in the right frontal, superior parietal, and bilateral occipital regions, indicates a high prior parasitic burden that managed to overcome maternal immune tolerance (12, 17).

Perfect clinical mimicry of hypertensive disorders: The combination of epigastric pain together with photophobia and fulminant headache is an almost exclusive reflection of impending eclampsia. In this patient, epigastric pain was an infrequent, atypical manifestation of neurocysticercosis, probably attributable to a reflex autonomic response to the acute increase in intracranial pressure caused by bilateral occipital vasogenic edema, rather than to distension of the hepatic Glisson's capsule (6, 10, 18).

Seizures in areas of FLAIR suppression without diffusion restriction: On MRI biophysical characterization, the frontal cortical and subcortical lesions showed a hypointense signal on T1 and a hyperintense signal on T2, with complete FLAIR suppression and an elevated ADC map, confirming free fluid content consistent with a pure vesicular stage (9). The atypical feature lies in the fact that the generalized seizures were triggered not only by the inflammatory colloidal lesions, but by a “threshold effect”: the cumulative burden of multiple viable cysts in areas of high epileptogenic susceptibility (such as the frontal cortex) within a gravid brain with a physiologically lowered seizure threshold (4, 11, 19).

This multidisciplinary scenario highlights the importance of timely neurological and neuroimaging evaluation in pregnant patients with neurovisual manifestations. Early identification of the underlying structural etiology potentially reduces the risk of seizure recurrence and associated maternal-fetal complications (8, 9). Finally, optimized clinical management with levetiracetam demonstrated an excellent safety profile, ensuring maternal stability without teratogenic effects or repercussions on the neonate's immediate neurodevelopment, thereby allowing a successful term vaginal delivery (11, 12).

The absence of documented severe hypertension, a 24-h urine protein level below the diagnostic threshold for significant proteinuria, and the recurrence of seizures led to a broader etiological approach, considering neurological, structural, vascular, metabolic, and infectious causes (20–22).

### 3.2 Diagnostic approach

In the emergency department, comprehensive clinical evaluations were performed, including general, neurological, and obstetric assessments, complemented by laboratory analyses and imaging studies. As part of the initial diagnosis, clinical findings consistent with an acute neurological and obstetric condition were identified, characterized by generalized tonic-clonic seizures, severe headache, epigastric pain, and blurred vision, accompanied by tongue biting and a postictal state on physical examination.

Laboratory tests showed positive dipstick proteinuria (2+), with a urine protein/creatinine ratio of 0.58 and a 24-h urine protein of 223.88 mg. Liver and kidney function tests remained within normal parameters. Blood pressure levels recorded in the medical records ranged from 90/60 to 126/64 mmHg; therefore, alternative causes were investigated to determine the origin of the symptoms, and neuroimaging studies were requested.

Additional serological tests included TORCH testing (toxoplasmosis, cytomegalovirus, rubella, herpes simplex) performed before 35 weeks of gestation with negative results, as well as a V.D.R.L. test performed at the beginning of pregnancy and at 37 weeks of gestation, both of which were nonreactive. Cerebrospinal fluid analysis was not warranted, as the presentation was not consistent with an acute infectious process. In addition, she had a negative O'Sullivan test performed at 28.2 weeks of gestation, and there was no evidence of immunological alteration during pregnancy, such as macrophage or T-lymphocyte activation, or elevated levels of TNF-α, IFN-γ, IL-1, and IL-6.

### 3.3 Therapeutic intervention

The patient's therapeutic management was multidisciplinary, initially aimed at stabilizing a seizure in the context of a pregnancy with suspected eclampsia and subsequent diagnosis of multilesional cerebral neurocysticercosis. This included anticonvulsant medications, antiparasitic therapy, and adjuvant corticosteroid treatment for seizures secondary to neurocysticercosis during pregnancy. In addition, expectant management was provided with continuous maternal-fetal monitoring.

In the initial phase, given the suspicion of hypertensive disorders of pregnancy with seizures, anticonvulsant treatment with magnesium sulfate was started, administered as a loading dose and maintenance dose, aimed at preventing and controlling eclamptic seizures. In addition, continuous maternal-fetal monitoring was performed due to the risk of maternal hypoxia, neurological compromise, and fetal distress associated with recurrent tonic-clonic seizures. As part of the initial neurological management, and given the persistent risk of seizures, levetiracetam 1,000 mg orally every 12 h was started (later adjusted to 750 mg orally every 12 h according to clinical progress), maintained as the baseline anticonvulsant treatment. Diazepam 5 mg intravenously was also administered as needed in cases of prolonged or recurrent tonic-clonic seizures.

As part of the etiological approach and the subsequent diagnostic confirmation of multilesional parenchymal cerebral neurocysticercosis, antiparasitic treatment with albendazole (600 mg orally for 14 days) was added, as monotherapy due to the unavailability of praziquantel in Ecuador, despite its inclusion in the country's National Basic Medicines List, together with dexamethasone 6 mg intravenously daily, aimed at reducing the inflammatory response associated with parasite death and lowering the risk of cerebral edema and seizure exacerbation (Table 2). Strict monitoring of maternal glucose and blood pressure was maintained throughout treatment, given the associated metabolic and neurological risks.

**Table 2:** Timing and rationale for treatment.

Treatment moment	Gestational age/postpartum	Treatment
First part of treatment	26.1 weeks of gestation	Levetiracetam 500 mg orally every 12 h
Second part of treatment	26.3 weeks of gestation	Dexamethasone injectable solution 6 milligrams intravenously every 12 h
Third part of treatment	27.2 weeks of gestation	Levetiracetam oral solid 1,000 mg: administer 750 milligrams orally every 12 h
Follow-up period	Immediate postpartum	Levetiracetam 1,000 mg oral solid: administer, 1,000 mg orally every 12 h In case of a generalized tonic-clonic seizure lasting more than 5 min, or 10 min for focal seizures: diazepam 5 mg/1 ml parenteral liquid: administer 5 mg IV if necessary. Albendazole administer 600 mg orally every 12 h for 14 days, starting 17/04/2026. Dexamethasone 4 mg/ml: administer 6 mg IV daily, starting 17/04/2026
Last follow-up	Late postpartum (9 days)	Prednisone 6 mg oral solid: 6 mg orally daily until 01/05/2026, then 4 mg orally daily for 4 days from 02/05/2026 to 05/05/2026, then 2 mg orally daily for 4 days from 06/05/2026 to 09/05/2026, and then discontinue Albendazole: administer 600 mg orally every 12 h, taking into account the start date of 17/04/2026 Levetiracetam 1,000 mg oral solid: administer 1,000 mg orally every 12 h

Timing and rationale for treatment.

During the clinical course, no major changes were needed to the anticonvulsant regimen, with levetiracetam maintained as the first-line drug due to an adequate response and the absence of new seizures. However, the treatment plan was progressively expanded based on the neuroimaging findings, shifting from an initial focus on eclampsia to the management of symptomatic epilepsy secondary to neurocysticercosis, which justified the addition of antiparasitic and corticosteroid therapy. The patient tolerated treatment well, with no serious adverse effects recorded during hospitalization.

## 4 Patient perspective

The patient expressed understanding and acceptance of the diagnosis and of the treatment plan proposed by the neurology and obstetrics departments. During follow-up, she reported progressive improvement of her headaches and the absence of new seizures after starting anticonvulsant treatment. She also expressed satisfaction with the multidisciplinary care she received and the information provided about the safety of breastfeeding during treatment. The favorable course of the pregnancy and, subsequently, of the postpartum period contributed to a positive perception of the care received.

## 5 Limitations

Despite the favorable outcome for both mother and newborn, the analysis of this case presented several limitations that should be considered when interpreting its results and conclusions. First, the marked similarity between the clinical manifestations of severe preeclampsia/eclampsia and neurocysticercosis caused an unavoidable delay in identifying the true etiology of the clinical picture. This situation prompted the initiation of magnesium sulfate treatment as a first-line therapeutic measure, given the high suspicion of eclampsia, an approach fully justified by the need to preserve maternal and fetal life. However, the definitive diagnosis corresponded to a parasitic disease of the central nervous system. This entity is uncommon during pregnancy and has few reports in the scientific literature as a differential diagnosis of hypertensive disorders of pregnancy.

Additionally, the limited availability of scientific evidence from similar cases made it difficult to compare clinical findings and to make decisions based on specific recommendations, thereby requiring individualized management of the patient through a multidisciplinary approach. The low frequency of this clinical association represents a significant limitation for establishing widely applicable diagnostic or therapeutic algorithms.

Furthermore, since this is a case report of a single patient, the findings cannot be extrapolated to the general population, nor do they allow for the establishment of causal relationships or definitive recommendations for clinical practice. Likewise, it was not possible to make comparisons with a control group or to evaluate different therapeutic strategies, limitations inherent to this type of study design.

Finally, it should be considered that epidemiological characteristics, personal history, access to neuroimaging studies, and the availability of specialized diagnostic resources may vary between institutions and regions, influencing the timeliness of diagnosis and therapeutic approach. Despite these limitations, this case highlights the importance of maintaining a high index of clinical suspicion and performing a thorough differential diagnosis in pregnant women with neurological manifestations, especially in areas endemic for neurocysticercosis, to optimize timely treatment and improve maternal and perinatal outcomes.

## 6 Conclusions

**Figure 4:**
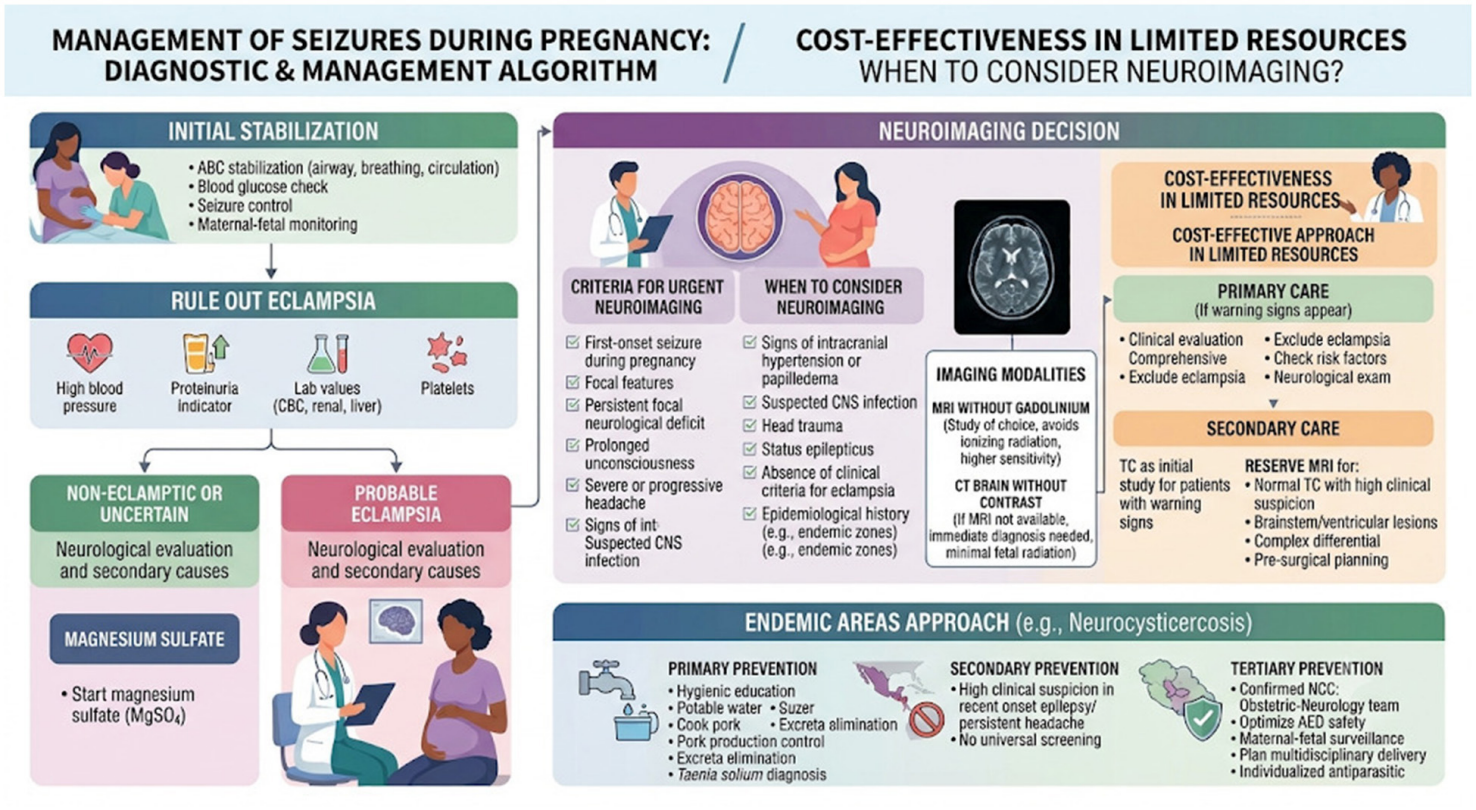
Clinical algorithm for management of seizures during pregnancy.

This case highlights that not all seizures during pregnancy are due to eclampsia, even in patients with a history of or risk factors for hypertensive disorders of pregnancy. In regions where Taenia solium infection is common, neurocysticercosis should be considered in the differential diagnosis of pregnant women with seizures, especially when these persist despite adequate treatment with magnesium sulfate. The lack of the expected clinical response should alert the medical team to a possible underlying neurological cause and prompt a more comprehensive evaluation, including neuroimaging. In addition, this case underscores the importance of international obstetric guidelines that recognize the role of parasitic infections of the central nervous system as potential mimickers of eclampsia in endemic areas, thereby promoting more timely diagnosis and comprehensive management, contributing to improved maternal and fetal outcomes. A clinical algorithm is recommended for the management of seizures during pregnancy (Figure 4).

## Data Availability

The original contributions presented in the study are included in the article/Supplementary material, further inquiries can be directed to the corresponding author.
